# Simultaneous Open Fractures of Both Patellae: A Case Report and Review of the Literature

**DOI:** 10.7759/cureus.110103

**Published:** 2026-06-02

**Authors:** Reda Bahij, Ayoub Daoudi, Mohammed-Reda Fekhaoui, Omar Aguenaou

**Affiliations:** 1 Department of Trauma and Orthopaedics, Faculty of Medicine, Ibn Sina University Hospital, Mohammed V University of Rabat, Rabat, MAR

**Keywords:** bilateral patellar fracture, case report, extensor mechanism, open fracture, orthopedic trauma, tension band wiring

## Abstract

We present the case of an 18-year-old male who presented following a high-energy motor vehicle accident with direct trauma to both flexed knees. Clinical examination revealed bilateral open anterior knee wounds with exposed bone fragments and intact distal neurovascular status. Plain radiographs confirmed bilateral displaced transverse patellar fractures (Gustilo-Anderson type II).

The patient underwent urgent single-stage open reduction and internal fixation of both patellae under general anesthesia. Thorough debridement and pulsed-saline lavage were performed bilaterally, followed by anatomical reduction and tension-band wiring fixation. The medial and lateral retinacula were repaired bilaterally. Postoperative radiographs confirmed satisfactory reduction. Early protected range-of-motion rehabilitation was initiated, and final follow-up at 16 months demonstrated uncomplicated healing, with full active bilateral knee extension (0°), flexion of 135° bilaterally, and a Lysholm Knee Score of 95/100.

Simultaneous bilateral open fractures of the patellae represent a rare and severe orthopedic injury, disrupting both extensor mechanisms while introducing the immediate infectious risks associated with open contamination. Bilateral simultaneous patellar fractures are rare, and open bilateral injuries are even more exceptional. We report a case of this uncommon trauma pattern and review the relevant literature.

## Introduction

The extensor mechanism of the knee is a complex functional unit comprising the quadriceps muscle and tendon, the patella, the patellar retinaculum, the patellar tendon, and the tibial tuberosity [1,2]. Its disruption, whether through fracture or tendon injury, results in the inability to achieve or maintain active knee extension [2]. The patella, the largest sesamoid bone in the body, acts as a mechanical lever arm within this system, increasing the moment arm of the quadriceps and thereby enhancing the efficiency of knee extension [3,4].

Patellar fractures account for approximately 1% of all skeletal fractures and occur predominantly in a unilateral distribution. Bilateral simultaneous patellar fractures are exceedingly rare, representing less than 3% of patellar fractures, and have been reported most commonly in association with dashboard injuries, direct trauma, metabolic bone disease, or pathological conditions [5,6]. Fractures of the patella within the extensor mechanism complex have been reported to occur six times more frequently than injuries to the patellar or quadriceps tendons [2]. Among bilateral patellar fractures, the transverse pattern is the most frequently observed type, with a higher prevalence in males aged 15 to 65 years [5].

Open bilateral patellar fractures following high-energy trauma are an even rarer subtype, combining the challenges of bilateral extensor mechanism reconstruction with the immediate infectious risks of open wound contamination. We report a case of simultaneous, symmetric open transverse fractures of both patellae in a young male following a motor vehicle accident, managed successfully with urgent single-stage fixation, and provide a review of the relevant literature.

## Case presentation

An 18-year-old male with no significant past medical history presented to the Emergency Department following a high-energy motor vehicle accident. He had sustained direct trauma to both flexed knees, resulting in isolated open injuries of both patellae, with a 4 cm laceration over the anterior left knee and a 3.5 cm laceration over the right. On physical examination, bilateral open wounds were present over the anterior knees, with exposed bone fragments visible through the lacerations, consistent with Gustilo-Anderson type II open fractures. Both knees demonstrated anterior swelling and tenderness. The patient was unable to perform active knee extension on either side. Distal neurovascular status was intact bilaterally, and no other significant injuries were identified on Advanced Trauma Life Support (ATLS) assessment.

**Figure 1:**
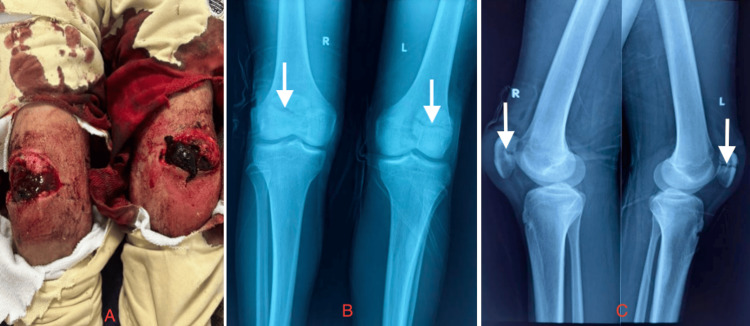
Clinical image and radiographs of bilateral open patellar fractures. (A) Clinical photograph showing open fractures of both patellae, with soft-tissue defects and exposed bone fragments. (B, C) Anteroposterior and lateral radiographs of both patellae demonstrating bilateral displaced patellar fractures, with the white arrows indicating the fracture line.

Plain radiographs of both knees in anteroposterior and lateral projections confirmed bilateral displaced transverse patellar fractures (Figure 1). The simultaneous, isolated, symmetric, and open nature of these fractures represents an exceptionally rare injury pattern, with very few comparable reports in the literature [5,7]. In the Emergency Department, both knees were immobilized with above-knee back slabs, intravenous antibiotic prophylaxis was commenced, and the patient received tetanus prophylaxis.

The patient was taken to the operating theatre on the day of admission. Under general anesthesia, bilateral surgical fixation was performed in a single session. Bilateral midline longitudinal incisions were made sequentially over each patella. Each fracture site underwent thorough debridement and pulsed-saline lavage in accordance with open fracture management principles.

**Figure 2:**
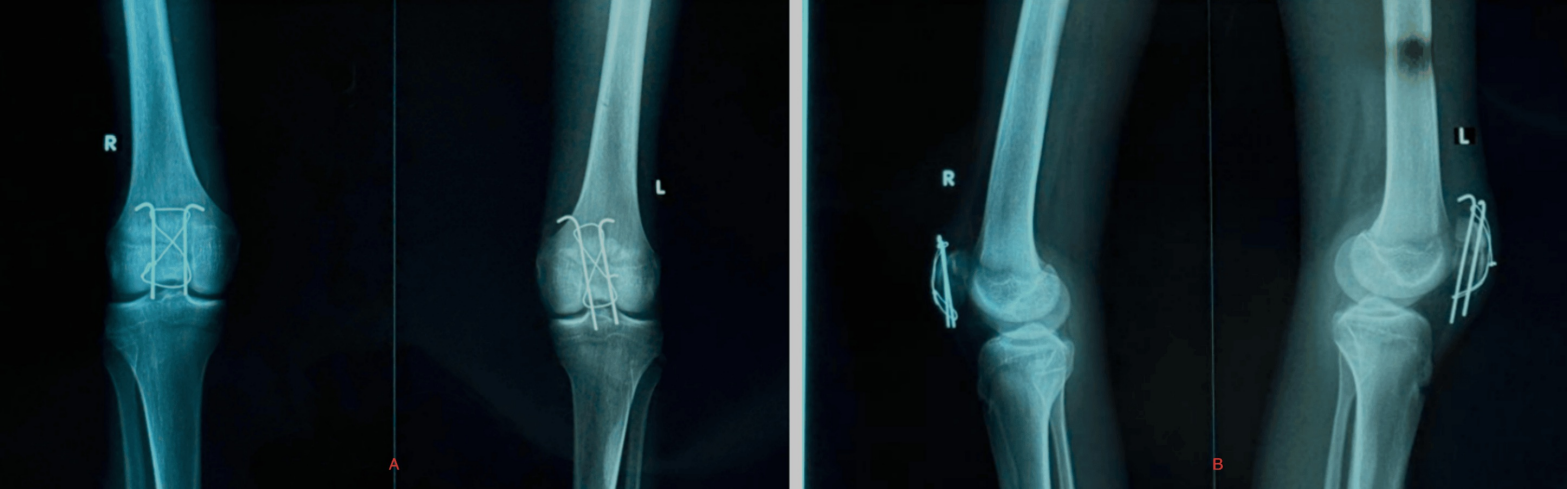
Postoperative anteroposterior radiographs of both knees confirming anatomical reduction and satisfactory bilateral tension-band wiring fixation of the patellae. The right knee (R) and left knee (L) are labeled accordingly.

Following adequate debridement, the fractures were anatomically reduced and stabilized on each side using the AO tension-band wiring technique. This involved the placement of two parallel Kirschner wires across the fracture site, followed by the application of a figure-of-eight tension-band wire, converting tensile quadriceps forces into compressive forces at the fracture surface and enabling early mobilization [5,8,9]. The medial and lateral retinacula were repaired bilaterally to restore extensor mechanism continuity and ensure appropriate patellar tracking. Wound closure was performed over suction drains bilaterally. Postoperative plain radiographs confirmed anatomic reduction and satisfactory hardware placement on both sides (Figure 2).

The postoperative course was uneventful. Wound healing progressed without signs of infection, wound dehiscence, or hardware complications. Drains were removed at 48 hours. Intravenous antibiotic prophylaxis was continued for 48 hours postoperatively and then transitioned to oral therapy.

Early rehabilitation was initiated on postoperative day 2 using bilateral removable knee braces set at 0-30 degrees of motion. Range of motion (ROM) was progressively increased according to a structured physiotherapy protocol: 0-60 degrees at four weeks and 0-90 degrees at six weeks. The patient tolerated bilateral therapy well. Partial weight-bearing with crutch assistance was permitted at three weeks postoperatively, with progression to full weight-bearing at seven weeks following radiographic evidence of early consolidation.

At final follow-up, full active extension was present bilaterally without extensor lag. Progressive recovery of knee flexion and functional capacity was documented on both sides, with no implant-related complications. The patient was followed for a total of 16 months, at which point the final functional assessment was performed. At final follow-up, active knee flexion reached 135° bilaterally, with full active extension (0°) and no extensor lag. The Lysholm Knee Score was 95/100, indicating excellent functional recovery. 

## Discussion

The patella, as the largest sesamoid bone in the body, serves as the central structural element of the knee extensor mechanism, functioning as a lever arm that enhances quadriceps efficiency and optimizes the moment arm for knee extension [3,4]. Bilateral simultaneous fractures of the patellae are exceedingly rare, accounting for less than 3% of all patellar fractures, and have been attributed to direct trauma, indirect muscular forces, stress mechanisms, or pathological bone conditions [5,6]. The first documented case of bilateral simultaneous patellar fractures is attributed to Desault in 1817 [10]. Among all reported bilateral cases, a recent systematic review identified that 97.4% involved closed injuries, with open bilateral fractures accounting for only 2.6% of cases [11].

There has been no major systematic review since Steinke's landmark 1913 compilation of historical cases until Nkosi et al. (2025) identified 19 patients across 18 studies in the English-language literature from 1913 to 2023 [11].

The most comprehensive synthesis of this topic to date is the systematic review by Nkosi et al. (2025), which identified only 19 patients with bilateral simultaneous patellar fractures across 18 published studies spanning from 1913 to 2023, with a male predominance (13/19) and a mean age of 42.1 years [11]. The present case - a bilateral open injury in an 18-year-old male - falls outside the previously described spectrum in both age and open fracture classification, further underscoring the exceptional rarity of this injury pattern.

Most historically reported traumatic bilateral cases involved closed fractures or asymmetric injury patterns. Recently, Nkosi and Klaas (2026) reported a 43-year-old male who sustained bilateral simultaneous patellar fractures following direct blunt trauma, treated with a hybrid fixation technique using cannulated screws augmented with fibre tape; their patient achieved fracture union but had a Knee Injury and Osteoarthritis Outcome Score (KOOS) of only 47% following implant removal, highlighting that functional outcomes in bilateral injuries can be suboptimal [12]. Similarly, Ozturk et al. (2023) reported bilateral isolated traumatic patellar fractures in a 27-year-old male following a motor vehicle accident, managed successfully with Kirschner wire fixation [13]. Several prior case reports have described bilateral traumatic patellar fractures following road traffic accidents managed with tension band wiring [7-9], but the present case adds an even rarer combination: bilateral symmetry, open fractures, and isolated patellar involvement in a young patient.

The mechanism of injury in our case - a high-energy direct trauma to both flexed knees in a motor vehicle accident - is consistent with previously reported traumatic bilateral cases [5,7,13]. Indirect bilateral fractures, by contrast, typically result from sudden violent quadriceps contraction with the knee in a flexed position and tend to produce transverse fracture patterns [9]. In our patient, the symmetry of the transverse fracture pattern suggests a combination of direct impact and transmitted tensile forces through the extensor mechanism.

Tension band wiring according to the AO principle remains the gold standard for displaced transverse patellar fractures without severe comminution. This technique exploits the biomechanics of the extensor mechanism by converting tensile forces at the anterior surface into compressive forces at the articular surface, promoting fracture consolidation and enabling early joint mobilization [5,8]. In the setting of open fractures, thorough debridement is a non-negotiable prerequisite before fixation. In our case, meticulous debridement and pulsed lavage were performed bilaterally prior to fixation, consistent with open fracture management principles, and contributed to the absence of infectious complications. Patellar plating, including hook plates and anatomical locking plates, has emerged as an important fixation alternative, particularly for comminuted patellar fractures, where tension band wiring may provide inadequate stability across multiple fragments; however, given the transverse, non-comminuted nature of our patient's fractures, standard tension band wiring provided sufficient biomechanical stability [8]. Heitzmann et al. (2021) demonstrated favorable outcomes using a hybrid approach combining screws and tension band wire for bilateral stress fractures [14]; however, for our patient's transverse fracture pattern without comminution, standard tension band wiring provided adequate stability.

Performing single-stage bilateral fixation offers important practical advantages: it limits total anesthetic exposure, allows simultaneous restoration of both extensor mechanisms, and facilitates a coordinated symmetric rehabilitation program, avoiding the asymmetric recovery that can complicate staged procedures. Nkosi and Klaas (2026) noted that delayed mobilization and quadriceps weakness are key challenges in bilateral patellar fractures, underscoring the value of early protected motion protocols [12]. LeBrun et al. (2012), in a study of 40 patients undergoing operative fixation for patellar fractures, reported that patellar fractures typically yield poor functional outcomes at intermediate follow-up [15]; our case, with progressive bilateral recovery and no extensor lag, represents a favorable result within this context, likely attributable to the young age of the patient and the early initiation of rehabilitation.

Our search of the English-language literature did not identify any prior report of simultaneous bilateral open patellar fractures with the symmetric transverse pattern described here in an otherwise healthy young patient. This rarity underscores the importance of a high index of clinical suspicion, systematic ATLS assessment, and individualized surgical planning in managing complex bilateral trauma.

This report has several limitations inherent to the case report design, including the absence of a control group and the inability to generalize the findings. The follow-up duration was limited to 16 months, which may not capture long-term outcomes such as post-traumatic arthritis or implant-related complications. No validated functional outcome scores were formally administered, and quantitative ROM measurements at each follow-up visit were not systematically recorded. Future prospective studies or registries capturing bilateral patellar fractures would provide higher-level evidence to guide management.

## Conclusions

Simultaneous bilateral open patellar fractures represent an exceptionally rare injury pattern requiring prompt, systematic orthopedic management. Urgent debridement, single-stage anatomical reduction, and stable fixation with tension band wiring, combined with bilateral retinacular repair and early coordinated rehabilitation, can achieve excellent functional outcomes. This case contributes to the very limited literature on bilateral open patellar fractures and reinforces the applicability of core open fracture management principles even in the setting of complex bilateral extensor mechanism trauma. A high index of suspicion, thorough clinical and radiographic assessment, and strict adherence to surgical principles are essential to optimize outcomes in this rare injury pattern.
